# Viral Diversity and Epidemiology in Critically Endangered Yangtze Finless Porpoises (Neophocaena asiaeorientalis *asiaeorientalis*)

**DOI:** 10.1128/spectrum.00810-23

**Published:** 2023-06-05

**Authors:** Xin Zhao, Caijiao Dai, Shiyu Qian, Qing Tang, Lijuan Li, Yujiang Hao, Zhijian Zhou, Xingyi Ge, Cheng Gong, Junfa Yuan

**Affiliations:** a Department of Aquatic Animal Medicine, College of Fisheries, Huazhong Agricultural University, Wuhan, People’s Republic of China; b Hubei Engineering Research Center for Aquatic Animal Diseases Control and Prevention, Wuhan, People’s Republic of China; c The Key Laboratory of Aquatic Biodiversity and Conservation of the Chinese Academy of Sciences, Institute of Hydrobiology of the Chinese Academy of Sciences, Wuhan, People’s Republic of China; d College of Biology & Hunan Provincial Key Laboratory of Medical Virology, Hunan University, Changsha, People’s Republic of China; e Tian-e-zhou National Reserve for Lipotes Vexillifer, Shishou, People’s Republic of China; Changchun Veterinary Research Institute

**Keywords:** Yangtze finless porpoise, *Papillomaviridae*, codivergence, viral diversity

## Abstract

The Yangtze finless porpoise (YFP) (Neophocaena asiaeorientalis *asiaeorientalis*) is a critically endangered freshwater cetacean, with about 1,249 individuals thought to be left in the wild. However, viral entities and viral diseases of YFPs remain obscure. In this study, anal swabs for virome analysis were collected during the physical examination of YFPs in the Tian-E-Zhou Oxbow (TEO) *ex situ* reserve. A total of 19 eukaryotic viral species belonging to 9 families, including *Papillomaviridae*, *Herpesviridae*, *Picornaviridae*, *Picobirnaviridae*, *Caliciviridae*, *Retroviridae*, *Parvoviridae*, *Virgaviridae*, and *Narnaviridae*, and other unclassified viruses were identified based on metasequencing. Among these detected viruses, a novel herpesvirus (NaHV), two different kobuviruses (NaKV1-2), and six different papillomaviruses (NaPV1 to -6) were considered potential risks to YFPs and confirmed by PCR or reverse transcription-PCR (RT-PCR). Most YFPs sampled were found to harbor one or more kinds of detected viral genomes (52/58 [89.7%]). Surveillance results demonstrated that kobuvirus and herpesvirus displayed obvious age distribution and PVs showed significant gender difference in YFPs. According to species demarcation criteria in individual genera in *Papillomaviridae*, two novel species (referred to as *Omikronpapillomavirus 2* and *3*) and four novel isolates of PV were identified in YFPs. Further evolutionary analysis suggested that NaPVs would occupy the mucosal niche and that virus-host codivergence mixed with duplications and host-switching events drives the evolution of cetacean PVs. Divergence times of PVs in YFP and other cetacean reflect the incipient speciation of YFPs. In summary, our findings revealed the potential viral entities, their prevalence, and their evolutionary history in YFPs, which raises an important issue regarding effects of viral infection on the fitness of YFPs.

**IMPORTANCE** The Yangtze finless porpoise (YFP) is the only cetacean species in freshwater following the functional extinction of the baiji (*Lipotes vexillifer*). Health management, disease treatment, and other special measures are important for maintaining the existing YFP populations, especially in *in situ* and *ex situ* reserves. The discovery of potential viral entities and their prevalence in YFPs raises an important issue regarding the effects of viral infection on the fitness of YFPs and may contribute to the conservation of YFPs. The evolutionary history of papillomaviruses in YFP and other cetaceans reflects the phylogeny of their hosts and supports the status of incipient species, opening a window to investigate the evolutionary adaptation of cetaceans to freshwater as well as their phylogeny to remedy the deficiency of fossil evidence.

## INTRODUCTION

The Yangtze finless porpoise (YFP) (Neophocaena asiaeorientalis *asiaeorientalis*) is a critically endangered freshwater cetacean, with about 1,249 individuals thought to be left in the wild (1). Most studies support the subspecies status of the Yangtze finless porpoise in Neophocaena phocaenoides or *Neophocaena asiaeorientalis* (2). Population genomics evidence indicates that YFPs are reproductively isolated from other porpoise populations and considered a unique incipient species (3). Currently, the YFP is the only cetacean species in the Yangtze River following the functional extinction of baiji (Lipotes vexillifer) and is also geographically isolated from other members of *Neophocaena* (4). Continuous environmental protection steps combining *in situ* and *ex situ* reserve strategies are critical for YFP species conservation (5). As well, health management, disease treatment, and other special rescue measures are important for maintaining the existing YFP populations, especially in *in situ* and *ex situ* reserves.

Multiple studies have focused on the bacterial contents of YFP feces and skin (6, 7). Normal hematological and biochemical analyte reference range intervals and a health evaluation system for YFPs were also established (8). However, viral entities and viral diseases of YFPs remain obscure (9). Occasionally, an individual that has died of unknown causes is observed in the natural habitat or *ex situ* reserves, and an association of this death with viral agents may be possible. According to studies in several members of the order Cetacea, diverse viral agents, including 15 papillomaviruses, which caused mild to severe clinical consequences were discovered (10). In the context of the coronavirus disease 2019 (COVID-19) pandemic, the YFP virome, an as-yet-unsolved mystery, is an attractive subject.

Viruses are suspected as the most abundant biological entities due to the fact that they infect almost every species. Dynamic and long-standing coevolution between viruses and their hosts may shape ecosystem diversity. Endogenous retrovirus and bornaviruses, referred to as viral fossils, have been used to mirror the phylogeny of their hosts (11). By tracing the infection of retrovirus in the genomes of modern humans and archaic hominins, a lineage in which Denisovans are more closely related to Neanderthals than modern humans was suggested (12). Coincidentally, the evolution analysis of papillomaviruses and polyomavirus, called living fossils, has also been used to reflect their host evolutionary history (13). Papillomaviruses (PVs), belonging to the family *Papillomaviridae*, have been isolated from reptiles, fish, birds, and mammals (14). Two of the most distinctive characteristics of PVs are their genotype-specific host restriction and low mutation rate (15). Investigation of the putative viruses in YFPs and other cetaceans could contribute to understanding the phylogeny of YFPs as well as the evolutionary adaptation of cetaceans to freshwater.

The Tian-E-Zhou Oxbow (TEO), a successful example of an *ex situ* reserve used to translocate YFPs to protect the species from extinction, has received general appreciation from the International Union for Conservation of Nature (IUCN), International Wildlife Conservancy (IWC), and the international cetacean academic circle. According to census data collected in April 2021, the population has grown to over 100 individuals, which is about the 1/10 of the existing YFPs worldwide. In this study, we aimed to describe the taxonomic diversity of viral communities in individual YFPs sampled in TEO and found diverse potential viral agents, including novel herpesvirus, kobuvirus, and papillomaviruses, in YFPs, raising the important issue of the effects of viral infection on the fitness of YFPs.

## RESULTS

### Diverse viruses in YFPs.

**FIG 1:**
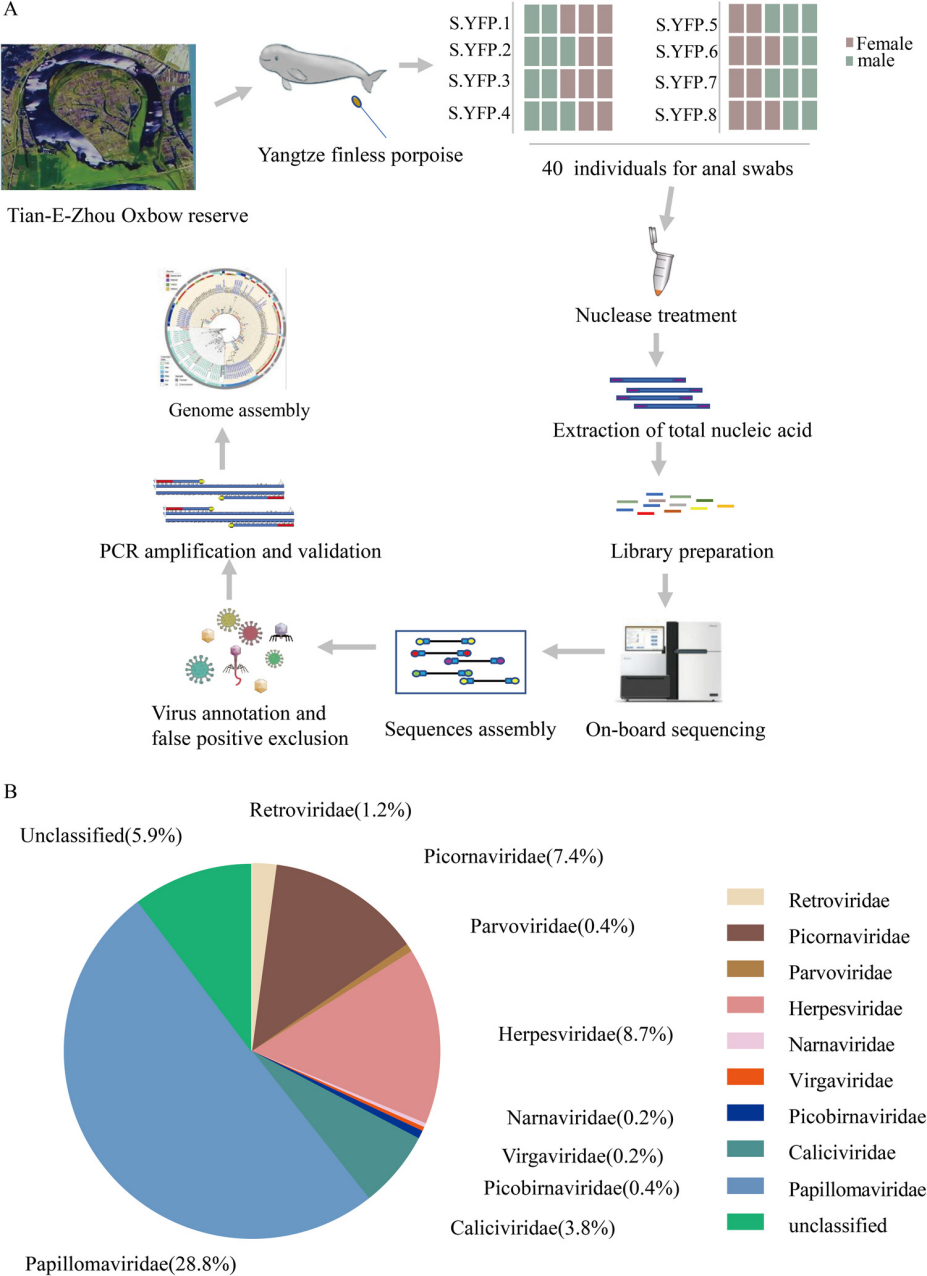
Composition of eukaryotic viruses detected in Yangtze finless porpoises. (A) Flow diagram of sampling and virus identification. (B) Composition of eukaryotic viruses detected in Yangtze finless porpoise fecal samples (family level). The relative abundance of viruses was calculated according to the percentage of viral contigs in all virus-like contigs. Nine families are indicated by different colors.

To explore the potential viral agents in YFPs, 58 anal swabs were obtained from YFPs in the TEO reserve when physical examinations were conducted. Every five anal swabs were pooled for one sample, and a total of 8 samples were used to extract nucleic acid and generate nucleic acid libraries (Fig. 1A). In total, 552,100,168 raw reads were obtained on the DNBSEQ-T7 platform from 8 libraries. After filtering of the low-quality raw reads, a total of 346,203 reads (0.08%) from the 397,373,817 clean data were annotated as viruses (Table 1). Clean reads were *de novo* assembled and compared with the viral reference database and GenBank nonredundant protein database using a BLASTx search with an E-value cutoff of <10^−5^. The overall composition of eukaryotic viruses detected in the feces from YFPs is presented in Fig. 1B. A total of 19 eukaryotic viral species belonging to 9 families, including *Papillomaviridae*, *Herpesviridae*, *Picornaviridae*, *Picobirnaviridae*, *Caliciviridae*, *Retroviridae*, *Parvoviridae*, *Virgaviridae*, and *Narnaviridae*, and other unclassified viruses were identified based on metasequencing and confirmed by PCR or reverse transcription-PCR (RT-PCR) and Sanger sequencing. Among these detected families, novel contigs related to *Papillomaviridae* showed relatively high abundance, at 28.8%.

**TABLE 1:** Numbers of reads obtained by sequencing and the percentage of filtered viral sequences from feces samples

Sample	No. of reads (PE)^*a*^			% viral reads
	Raw	Clean	Virus	
S.YFP.1	68,885,069	54,202,627	67,917	0.1253
S.YFP.2	67,481,400	51,231,896	23,315	0.0455
S.YFP.3	68,225,523	49,729,121	63,968	0.1286
S.YFP.4	67,184,566	51,745,049	124,286	0.2402
S.YFP.5	66,127,542	35,057,147	22,679	0.0647
S.YFP.6	79,051,590	59,984,881	31,959	0.0533
S.YFP.7	66,747,050	40,848,395	8,572	0.021
S.YFP.8	68,397,446	54,574,701	3,507	0.0064

a PE, paired-end.

### Prevalence of eukaryotic viruses in YFPs.

**FIG 2:**
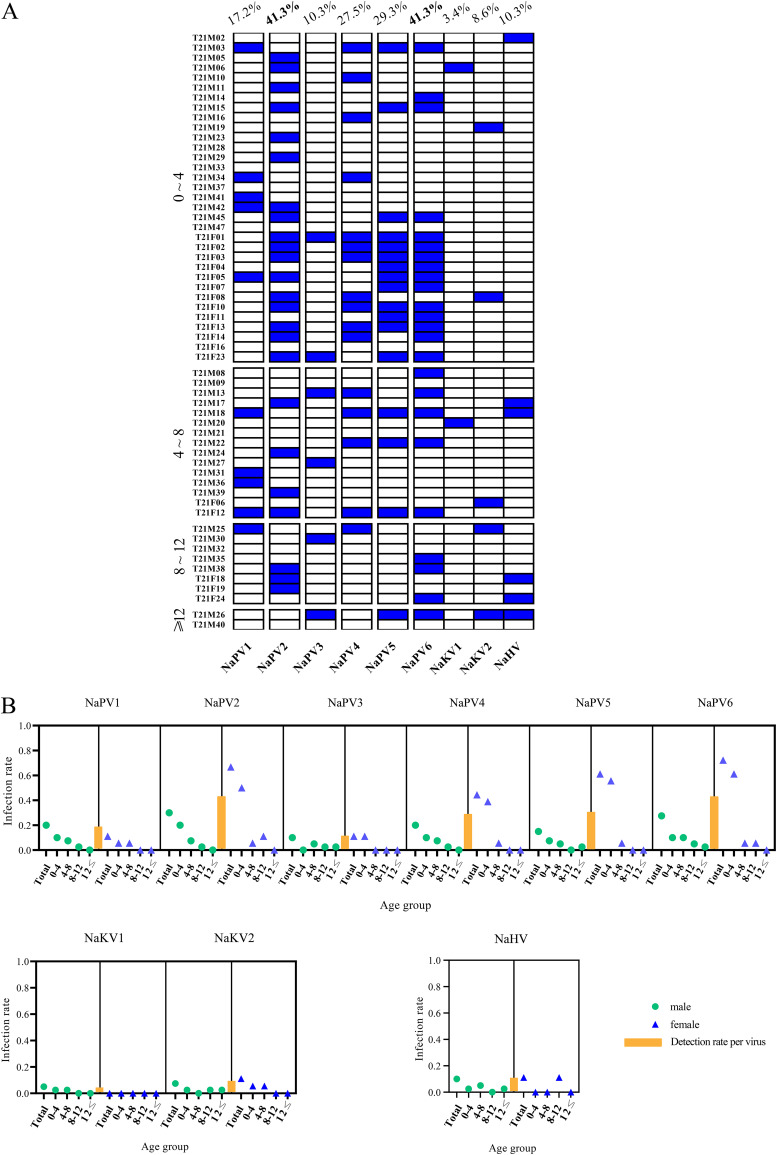
Prevalence of indicated viruses in Yangtze finless porpoises. (A) Overview of the indicated viruses in Yangtze finless porpoises. All individuals are displayed in age groups. Blue represents positivity for the detected virus. (B) Prevalence of indicated viruses, grouped by age and gender.

Among the eukaryotic viruses identified, a novel herpesvirus (NaHV), two different kobuviruses (NaKVs), and six different papillomaviruses (NaPV1 to -6) were considered to pose potential risks to YFPs. Based on sequences of the newly identified contigs, PCR or RT-PCR screening was conducted for prevalence investigation. All tested individuals were found to harbor at least one kind of virus (52/58 [89.7%]) with the exception of 6 YFPs. As shown in Fig. 2A, 10.3 to 41.3% of YFPs were papillomavirus positive, with the highest prevalence being seen with NaPV2 and NaPV4, while 9 YFPs were positive for more than four kinds of papillomaviruses. Seven YFPs were kobuvirus positive, with NaKV2 having the highest prevalence (5/58 [8.6%]). The prevalence of papillomaviruses in YFPs showed no obvious age distribution, while older YFPs (over 8 years old) showed higher rates of positivity for NaKV2 (2/10 [20%]) and NaHV1 (3/10 [30%]) than young YFPs. Higher prevalence of NaPVs was observed in the female populations than in the male population. For instance, the positivity rates of NaPV2, NaPV5, and NaPV6 reached 66.67%, 61.11%, and 72.22% in female YFPs, whereas they were 30.30%, 15.00%, and 27.50% in male YFPs (Fig. 2B). There was no obvious gender difference for the prevalence of kobuviruses or herpesvirus in YFPs.

### Molecular characterization of novel papillomaviruses in YFPs.

**FIG 3:**
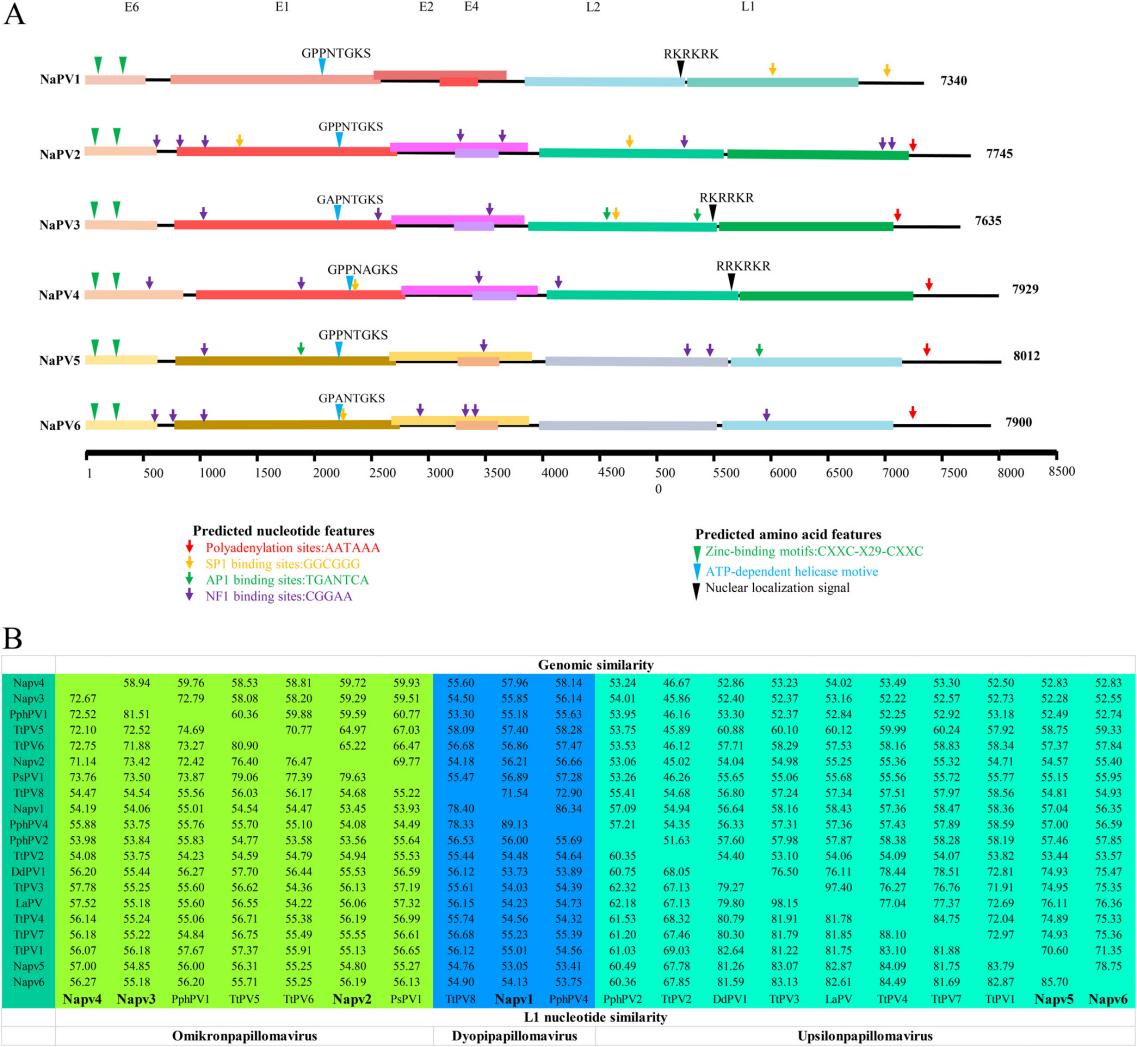
Molecular characterization of novel papillomaviruses identified in Yangtze finless porpoises. (A) The genomic structure of NaPVs. The length of each viral genome is indicated. The predicted ORFs, including E6, E1, E2, E4, L2, and L1, are shown with colored rectangles. Similar color schemes were used to indicate similar NaPVs. The predicted characteristic nucleotide sequence and putative motifs in each strain are indicated with colored arrows. (B) Percent similarity of the full-length genome (top) and L1 nucleotide sequence (bottom) from the six strains of papillomavirus identified in Yangtze finless porpoises (NaPVs) to cetacean papillomaviruses. Cetacean papillomaviruses from three genera are shown in different colors, and NaPVs identified in this study are shown in bold.

Based on Illumina sequencing and Sanger sequencing, six full-length genomic sequences of papillomaviruses, referred to as NaPV1 to -6, were recovered from YFP anal swabs. The complete genomic sequences of NaPVs varied from 7,340 to 8,012 bp, with GC contents of 45.63 to 48.78%. Like other identified papillomaviruses, the genomes of NaPVs contain 6 ORFs which encode 4 early proteins (E6, E1, E2, and E4) and 2 capsid proteins (L2 and L1) on the same strand of their double-stranded DNA (dsDNA). In contrast to the high-risk HPVs (HPV1, -16, and -17), a canonical E7 open reading frame (ORF) is missing in the genomes of NaPVs, which was similar to other reported cetacean PVs. Analysis of the genomic and deduced amino acid sequences revealed that many of the classic PV-specific elements were present in the 6 NaPVs (Fig. 3A). The deduced proteins of E6 ORFs in NaPVs genomes are 185 to 261 amino acids (aa) in length and contain two conserved zinc finger motifs with the sequence CXXC-X_29_-CXXC separated by 36 aa. NaPV1 harbors a PDZ domain binding motif in the C terminus of the E6 ORFs, which has been observed in high-risk genital HPVs, indicating the molecular similarities between NaPV1 and high-risk PVs (see Table S1 in the supplemental material).

Pairwise comparisons of L1 genes revealed 53.41 to 89.13% nucleotide identity among NaPV1 to -6. The highest nucleotide identity, 85.7%, was observed between NaPV5 and NaPV6, while they had 53.41 to 89.13% nucleotide identity to reported papillomaviruses in cetaceans, with the highest value being 89.13% between NaPV1 and PphPV4. When their full-length genomic sequences were aligned, the identity ranged from 49.56% to 78.75% among NaPV1 to -6 and 45.02% to 97.40% among all identified cetacean papillomaviruses. In detail, NaPV1 shared 85.16% nucleotide identity with PphPV4 in the genus *Dyopipapillomavirus*, in which intragenus recombinations were proposed. NaPV2, NaPV3, and NaPV4 shared higher similarities to PVs in the genus *Omikronpapillomavirus* that were associated with genital lesions in cetaceans. However, NaPV3 shared 72.79% nucleotide identity with PphPV, but NaPV2 and NaPV4 shared less than 70% nucleotide identity with any other PVs in the genus *Omikronpapillomavirus*. NaPV5 and -6 shared 78.75% identity with each other and 75% with *Upsilonpapillomavirus 1* in the genus *Upsilonpapillomavirus* (Fig. 3B). According to the species demarcation criteria in the family *Papillomaviridae*, 2 novel species (NaPV2 and NaPV4) and 4 new isolates of PVs were identified in YFPs.

### Phylogenetic analysis of papillomaviruses in YFPs.

**FIG 4:**
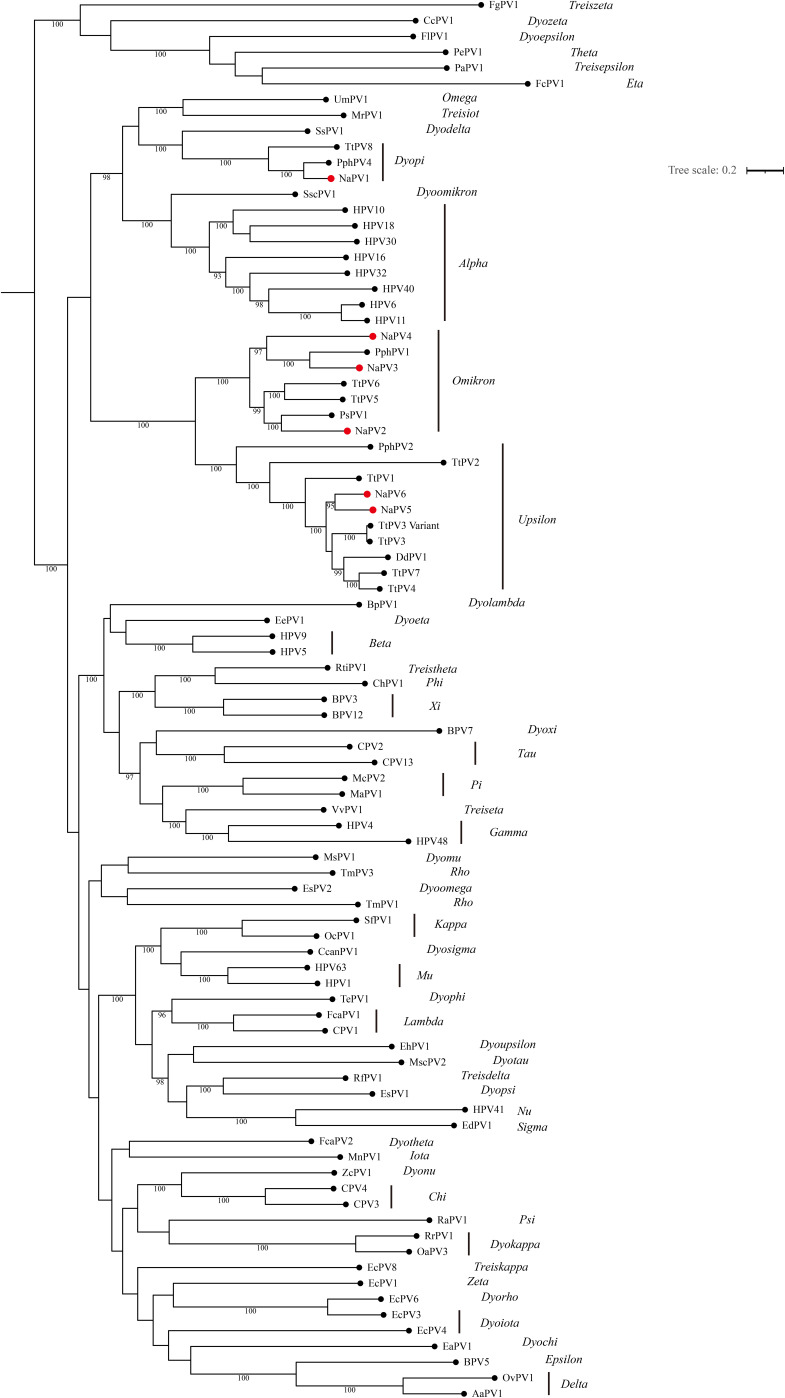
Maximum-likelihood phylogenetic tree based on concatenated E1, E2, L2, and L1 amino acid sequences of PVs. Sequences from the six new papillomaviruses detected in this study (NaPVs) were aligned with reported cetacean papillomaviruses and other representative species from each papillomavirus genus. The phylogenetic tree was constructed using the LG+F+R9 model through IQ-tree. The number on the branch indicates the Bayesian posterior probability, and values higher than 90 are shown. NaPVs are highlighted with red dots.

For classification purpose, phylogenetic trees were constructed for the concatenated E1, E2, L2, and L1 nucleotide sequences, based on multiple-sequence alignments, including reported PV type species and the novel YFP PVs. As shown in Fig. 4, the PVs cluster according to the previously defined genera and all cetacean PVs, including NaPVs, can be observed in three genera, *Dyopi-*, *Omikron-*, and *Upsilonpapillomavirus*. NaPV1 was clustered with PphPV4 and TtPV8, which were classified as *Dyopipapillomavirus 1*, while NaPV2 to -4 were clustered with *Omikronpapillomavirus 1* in the genus *Omikronpapillomavirus*. NaPV5 shows closer clustering relationships with NaPV6 and *Upsilonpapillomavirus 1* than *Upsilonpapillomavirus 2* and *3* in the genus *Upsilonpapillomavirus*. Combining these data with the alignment of full-length genomes and topology of phylogenetic trees, we propose that (i) NaPV1 can be classified as a novel isolate of *Dyopipapillomavirus 1* in the genus *Dyopipapillomavirus*; (ii) NaPV2 and NaPV4 can be classified as two novel species (*Omikronpapillomavirus 2* and *3*), while NaPV3 can be classified as a novel isolate of *Omikronpapillomavirus 1* in the genus *Omikronpapillomavirus*; and (iii) NaPV5 and NaPV6 should be classified as novel isolates of *Upsilonpapillomavirus 1* in the genus *Upsilonpapillomavirus*.

### Evolutionary history of papillomaviruses in YFPs.

**FIG 5:**
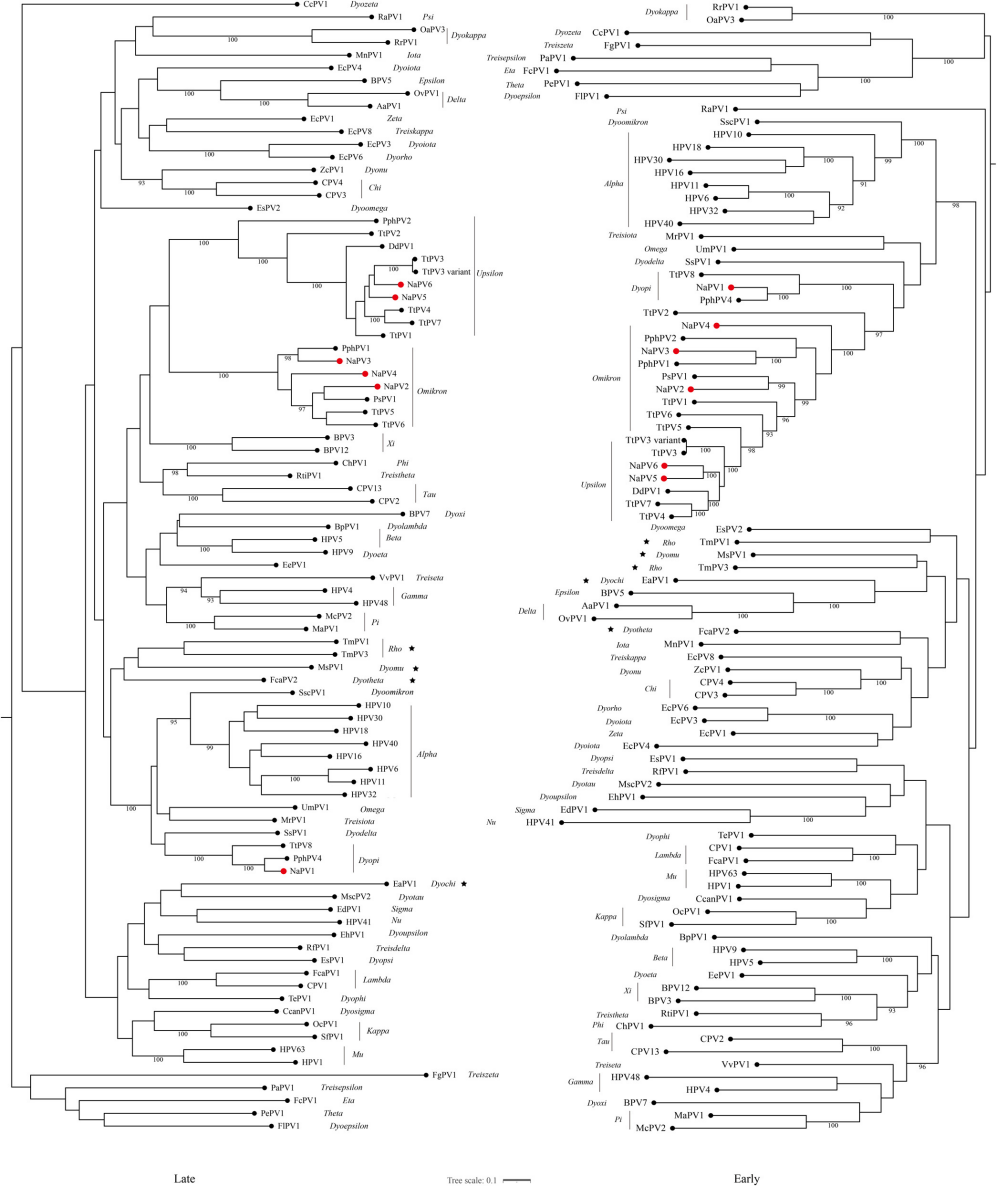
Maximum-likelihood phylogenies based on the E1 and L1 nucleotide sequences. (A) NaPVs and other representative papillomaviruses were included to constructed maximum-likelihood phylogenetic trees based on the E1 and L1 nucleotide sequences. The phylogenetic tree was constructed using the GTR+F+R6 model through IQ-tree. The number on the branch indicates the Bayesian posterior probability, and values higher than 90 are shown. NaPVs are highlighted with red dots. Asterisks indicate the major incongruence of the topology between the phylogenetic trees based on different parts of the genome of PVs.

Separate maximum-likelihood phylogenetic trees were constructed for the L1 and E1 nucleotide sequences. The topologies of phylogenetic trees based on different parts of the genome of PVs are incongruent (Fig. 5). The most significant characteristic is that all cetacean PVs and *Dyodeltapapillomavirus* members were monophyletic in early-gene analysis, whereas NaPV1 and *Dyopipapillomavirus* members were clustered with *Treisiotapapillomavirus*, which was far distant from other cetacean PVs in late-gene analysis. Similar jumps between different crown groups from two phylogenetic trees were also observed from *Iota*-, *Tau*, and *Chipapillomavirus*. Based on the E1 phylogenetic trees, mucosal high-risk viruses (human papillomavirus 16 [HPV16] and [HPV18]), mucosal low-risk viruses (HPV11 and HPV40), and viruses associated with cutaneous lesions (HPV41, HPV5, HPV63, and HPV4) are grouped separately (16). NaPVs and other cetacean PVs were clustered with *Alphapapillomavirus*, representing the mucosal tropism in the E1 phylogenetic trees, while most of the PVs from *Rho-*, *Delta-*, *Dyochi-*, *Nu-*, *Beta-*, and *Gammapapillomavirus* were clustered together and represented the cutaneous niche. We inferred that NaPVs would occupy the mucosal niche.

**FIG 6:**
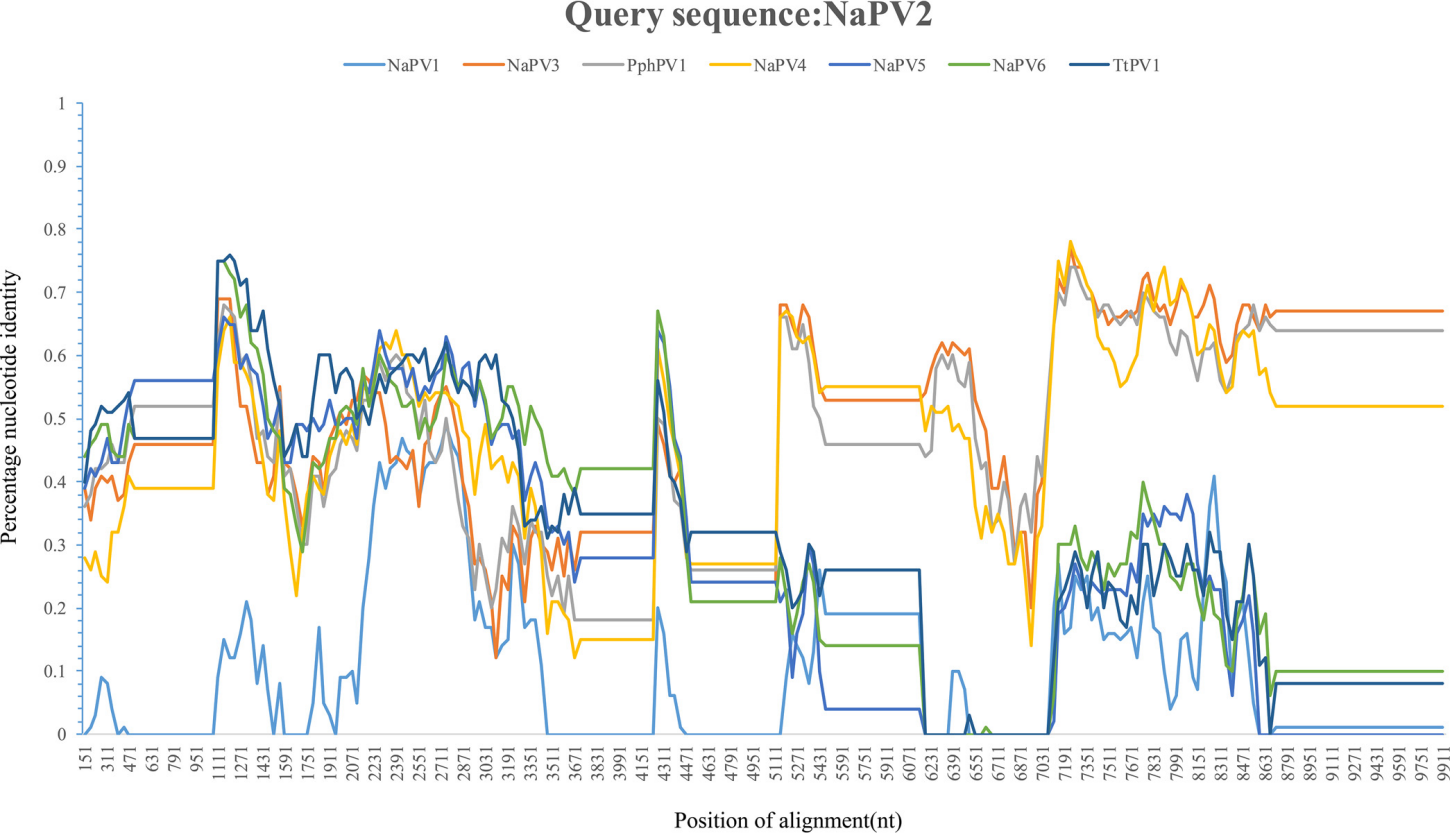
Recombination analysis of the genomes of novel papillomaviruses in identified in Yangtze finless porpoises. Simplot analysis was performed on a complete genome alignment of the Yangtze finless porpoises PVs PphPV1 and TtPV1. The NaPV2 sequence was used as the query, and the analysis was performed with a window size of 300 nucleotides and a step size of 40 nucleotides. The positions in the alignment are correlated with the positions on the NaPV2 genome.

Recombination played a role at specific moments throughout PV evolution, indicating that PVs and their hosts did not follow identical evolutionary paths (17). Using RDP4, a unique recombination event was detected across both the early and late regions of the PV genomes, which was supported by 7 recombination detection algorithms. In this event, the beginning and ending breakpoints correspond closely with the full length of the late region of the genomes, spanning a length of over 3 kb. Potential recombinant sequences of NaPV2 derived from the major parent TtPV1 and the minor parent PphPV1. The BootScan algorithm implemented in Simplot v3.5.1 was used to verify this recombination and indicated that the closest relatives of NaPV2 were TtPV1 in the early genes and PphPV1 in the late genes (Fig. 6).

The *dN*/*dS* ratios (ratios of nonsynonymous to synonymous mutations) and the presence of positively selected sites for the E6, E1, E2, L2, and L1 ORFs from cetacean PVs, including NaPVs, were calculated with the MEME, FUBAR, SLAC, and FEL algorithms for codon substitution (Table 2). MEME and FEL gave a higher number of positively selected codons than other two methods. More positively selected positions were detected in the early genes (E1 and E2) than in late genes. Most of the positions identified as being positively selected were unique to a single method. However, five codon positions in E2 were detected by all four methods (235, 243, 369, 400, and 402).

**TABLE 2:** Presence of positively selected sites in the E6, E1, E2, L2, and L1 ORFs of NaPVs

ORF	No. of sites (amino acid position[s]) calculated by^*a*^:			
	MEME (*P *< 0.1)	FUBAR (*P *> 0.9)	SLAC (*P *< 0.1)	FEL (*P *< 0.1)
E6	20 (11, 59, 63, 77, 83, 95, 103, 119, 150, 159, 160, 162, 163, 167, 177, 187, 239, 242, 290, 295)	1 (241)	None	1 (242)
E1	50 (4, 5, 15, 23, 28, 47, 66, 76, 106, 109, 127, 160, 163, 166, 167, 171, 188, 190, 191, 195, 206, 207, 211, 220, 238, 240, 252, 253, 271, 272, 291, 317, 353, 396, 415, 427, 442, 473, 478, 516, 528, 534, 537, 549, 555, 597, 615, 634, 637, 658)	3 (163, 380, 637)	7 (4, 71, 157, 173, 189, 200, 471)	12 (4, 23, 102, 142, 157, 173, 184, 204, 471, 630, 662)
E2	56 (5, 14, 22, 39, 60, 80, 88, 134, 140, 143, 150, 155, 158, 190, 192, 201, 206, 207, 225, 228, 230, **235**, 237, 239, **243**, 273, 306, 309, 310, 314, 317, 329, 343, 345, 349, 352, 360, 361, 368, **369**, 392, 393, 396, **400**, **402**, 404, 405, 408, 412, 414, 444, 450, 471, 475, 477, 483)	16 (134, 206, 230, **235**, **243**, 260, 310, 343, 346, 360, **369**, **400**, **402**, 404, 412, 471)	8 (**235**, **243**, 270, 279, 349, **369**, **400**, **402**)	25 (5, 92, 134, 206, 229, 230, **235**, 239, **243**, 260, 310, 329, 343, 349, 360, 365, **369**, 371, 392, 396, **400**, **402**, 404, 412, 471)
E4	11 (22, 61, 63, 102, 106, 114, 133, 206, 222, 250, 252)	2 (175, 179)	None	3 (22, 63, 133)
L1	17 (10, 35, **76**, 116, 161, 241, 299, 319, 324, 387, 391, 399, 462, 510, 525, 546, 555)	2 (**76**, 322)	1 (**76**)	1 (**76**)
L2	26 (7, 12, 25, 26, 31, 43, 62, 70, 94, 118, 154, 252, 268, 301, 313, 325, 329, 336, 458, 476, **481**, 493, 539, **50**, 600, 649)	None	None	2 (**481**, **540**)

a Numbers in parentheses are the amino acid positions in the corresponding proteins. Amino acid positions found to be positively selected are in bold.

### Divergence times of papillomaviruses reflecting the incipient speciation of YFPs.

**FIG 7:**
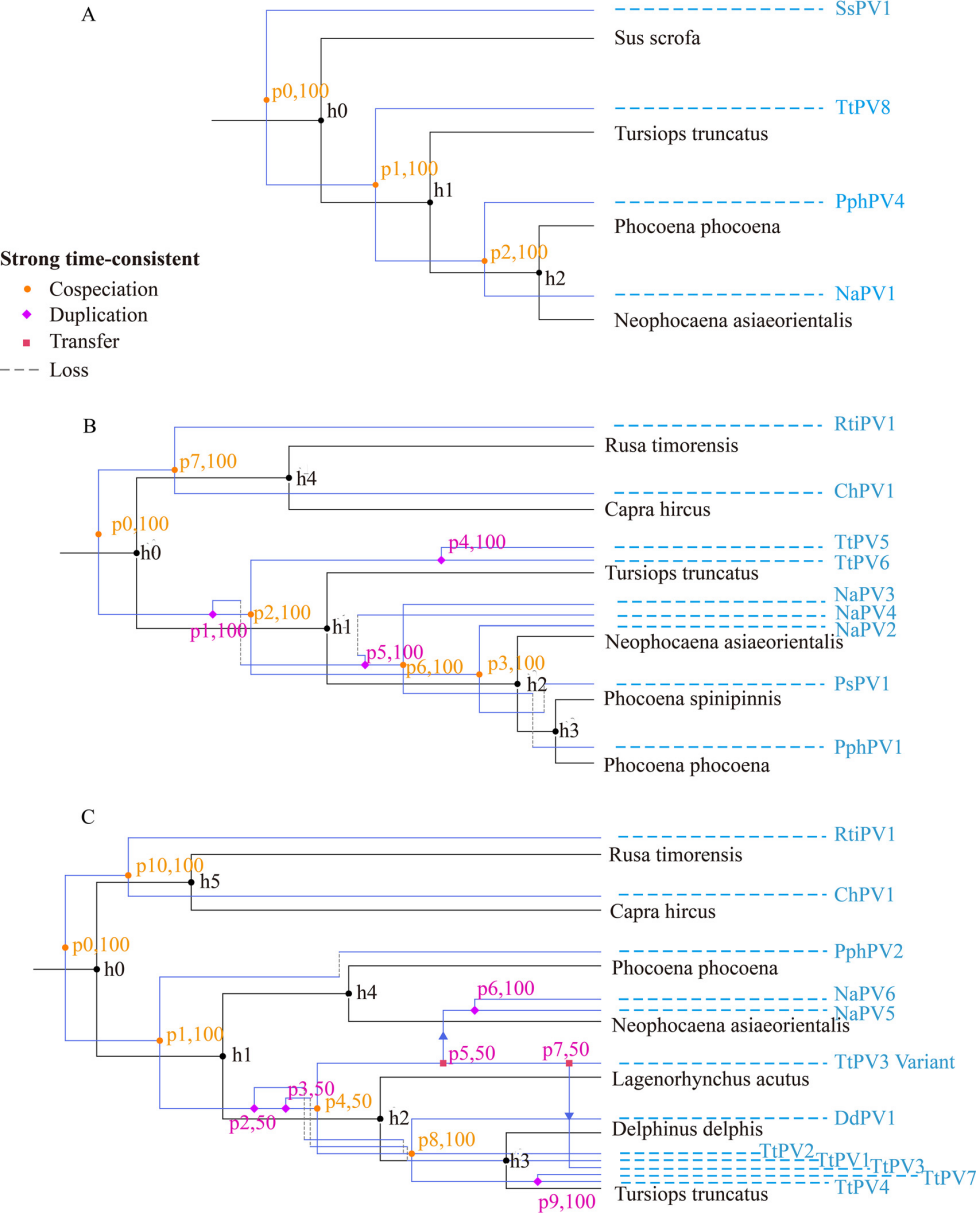
Cophylogenetic comparisons for cetacean papillomaviruses with their hosts. The blue phylogenies were reconstructed, representing the divergence of viral species according to the nucleotides of tandem E6-E1-E2-L2-L1 and mapped to the black phylogenies, which show the host divergence based on the mitochondrial full-length gene. (A) SsPV1 from *Dyodeltapapillomavirus* was used as the outgroup of all *Dyopipapillomavirus* viruses, and duplication cost and transfer cost were set to 1 and 2, respectively. (B and C) RtiPV1 from *Treisthetapapillomavirus* and ChPV1 from *Phipapillomavirus* were used as outgroups of all PVs from *Omikronpapillomavirus* and *Upsilonpapillomavirus*. The corresponding duplication and transfer costs for *Dyopipapillomavirus* and *Omikronpapillomavirus* are set to 1 and 2, respectively, and the corresponding replication and transfer costs for *Upsilonpapillomavirus* are set to 4 and 6.

Using the maximum-likelihood method, the polygenetic trees of all cetacean PVs and their hosts based on the concatenated E1, E2, L2, and L1 nucleotide sequences and full-length mitogenomes, respectively, were constructed. The cophylogenetic analyses of all cetacean PVs and their respective hosts were performed separately for *Dyopi-*, *Omikron*-, and *Upsilonpapillomavirus* by eMPRess considering five cost schemes for the events. The best-supported solution for *Dyopipapillomavirus* indicated cospeciations (Fig. 7A). However, 5 cospeciations, 3 duplications, and 4 losses were inferred for *Omikronpapillomavirus* (Fig. 7B). Inferred duplications between TtPV5 and TtPV6 and between NaPV2 and -3 and NaPV4 were observed. As well, there were 5 cospeciations, 4 duplications, 5 losses, and 2 host-switching events for *Upsilonpapillomavirus* (Fig. 7C). Notably, the two host-switching events were suggested to have occurred between Lagenorhynchus acutus and YFP and between *L. acutus* and Tursiops truncatus by the linkage of the TtPV3 variant. Therefore, the analyses indicated that virus-host codivergence mixed with duplications and host-switching events drives the evolution of cetacean PVs.

**FIG 8:**
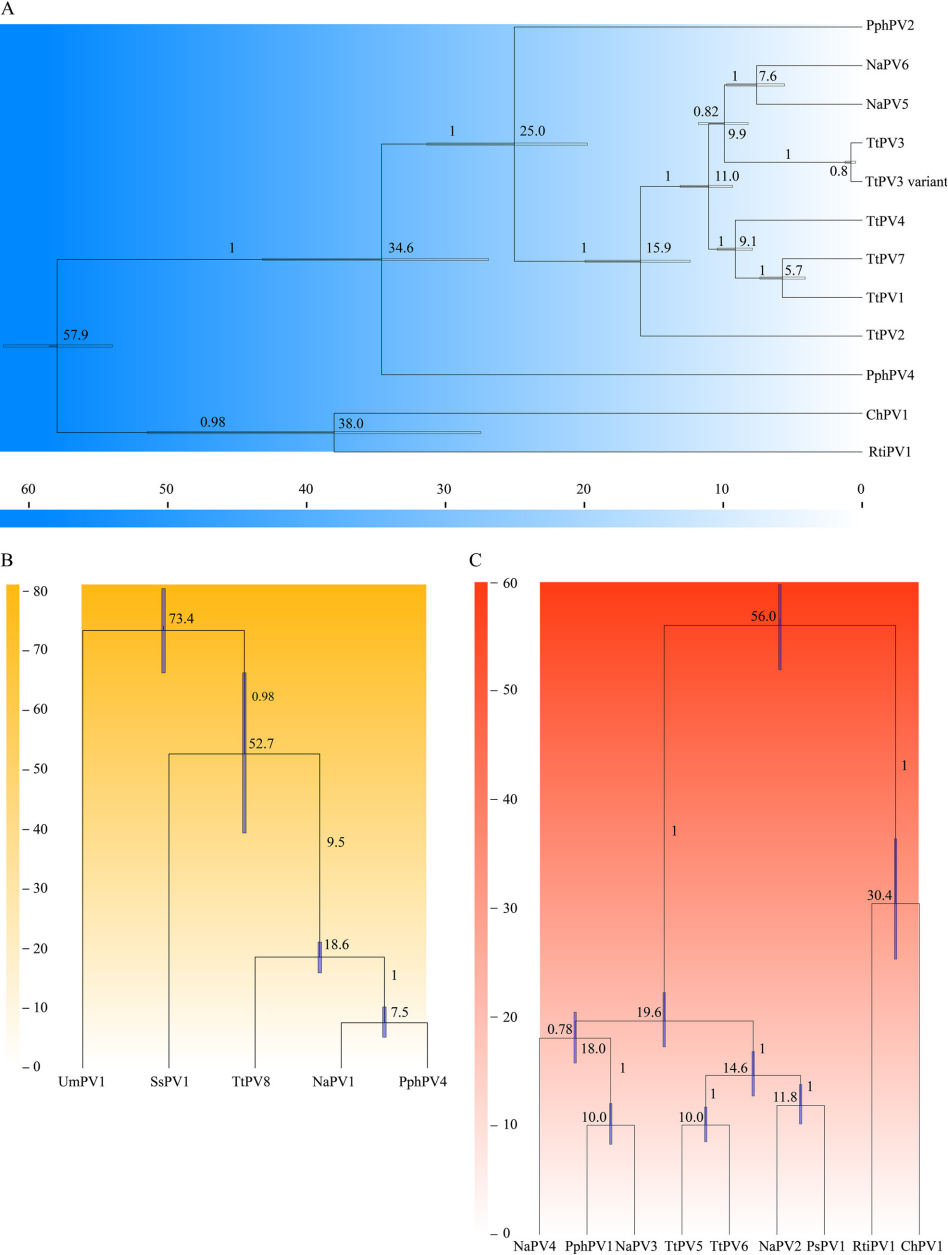
Divergence time estimation of cetacean papillomaviruses. A Bayesian MCMC method based on the relaxed-clock log-normal model was used to estimate divergence times. Times were calculated separately for each genus, *Upsilonpapillomavirus* (A), *Dyopipapillomavirus* (B), and *Omikronpapillomavirus* (C). Numbers above the nodes are the mean estimated divergence times (Mya). The bars represent the 95% HPD interval for the divergence times. Panels B and C show time on the *y* axis and phylogeny on the *x* axis.

Employing previously established PV evolution rates, a Bayesian statistical framework was used to estimate the divergence times of NaPVs from their most recent common ancestors (MRCAs) (18). As shown in Fig. 8A, the members of *Upsilonpapillomavirus*-*Phipapillomavirus* split from an MRCA around 57.9 million years ago (Mya) (95% highest posterior density [HPD], 54.0 to 61.8 Mya), which was consistent with the time frame (56 to 53 Mya) of the split between the Cetacea and their terrestrial artiodactyl ancestors. In contrast, the divergence time between them was 48.6 Mya (41.6 to 55.7 Mya; 95% HPD) according to the exponential time-dependent rate (TDRexp) clock model (Fig. S1A), which is later than the time of divergence between the Cetacea and their terrestrial artiodactyl ancestors. The species TtPV2, representing *Tursiops truncatus*, splits from an MRCA with the species around 34.6 Mya, corresponding to the first period of ocean restructuring (35 to 31 Mya), whereas most of the cetacean PVs diverged around the second period of ocean restructuring (13 to 4 Mya), reflecting the diversifying radiation of most extant species. In comparison, the time of divergence between NaPV1 and PphPV4 is around 7.5 Mya (5.1 to 10.2 Mya; 95% HPD) (Fig. 8B), reflecting the time of divergence of YFPs and Phocoena phocoena. *P. phocoena* and *Neophocaena phocaenoides* were indicated to diverge around 8.3 Mya (7.8 to 9.6 Mya; 95% HPD). When the TDRexp model was used, the time of divergence between NaPV1 and PphPV4 was 4.3 Mya (3.2 to 5.6 Mya, 95% HPD) (Fig. S1B). With the exception of NaPV4, other NaPVs diverged from other cetacean PVs later than 11 Mya. The phylogenetic tree suggested that NaPV4 diverged with the clade of PphPV1/NaPV3 around 18 Mya (Fig. 8C). The time of divergence of YFPs and *P. phocoena* and of *P. phocoena* and *N. phocaenoides* indicates the status of incipient species. The host-switching event between the hosts of TtPV3 and TtPV3 variants was estimated at 0.8 Mya (0.4 to 1.2 Mya; 95% HPD), which is significantly lower than the current consensus for the time to the MRCA (tMRCA) of their respective hosts, while another inferred transfer from the TtPV3 variant to NaPV5/6 was estimated at approximately 9.9 Mya (0.4 to 1.2 Mya; 95% HPD), indicating the ancient trace of evolution.

## DISCUSSION

Viral metagenomics has enabled the discovery of viral agents in humans, game animals, and rare wild animals (19–21). We first focused on the potential viral agents in endangered YFPs and revealed vastly diverse viral sequences as a tip of the iceberg. These findings could contribute to the healthy management of YFPs as well as the development of emergency rescue techniques.

During the physical examination of YFPs in the TZO reserve, anal swabs were collected from these YFPs for virome analysis. Not surprisingly, diverse viruses belonging to the families *Papillomaviridae*, *Herpesviridae*, *Picornaviridae*, *Picobirnaviridae*, and *Caliciviridae* were found in the feces from YFPs. Recently, members of *Picornaviridae*, *Picobirnaviridae*, and *Caliciviridae* were frequently discovered in diverse hosts, including giant pandas, tuna, and many other hosts, via high-throughput sequencing, which greatly expands the number and host range of these viruses according the reports of ICTV (22–24). By PCR or RT-PCR methods, most YFPs were found to harbor one or more kinds of viral genomes. According to the limited data, we inferred cautiously that kobuvirus and herpesvirus displayed obvious age distribution while PVs showed significant gender difference. Actually, α- and γ-herpesviruses show obvious age distribution, and older individuals have higher prevalence and morbidity. γ-herpesviruses, including Epstein-Barr virus (EBV) and Kaposi’s sarcoma-associated herpesvirus (KSHV), are associated with the human diseases infectious mononucleosis and Kaposi's sarcoma (25). *Kobuvirus*, belonging to the family *Picornaviridae*, was first established in 1999 and was recently found to be linked to diarrhea in calves and felines (26, 27). Considering that all YFPs tested showed no obvious clinical symptoms, we could deduce that the detected viruses posed a low direct risk to their hosts. However, increasing bodies of evidence indicated that low-pathogenicity viral agents could cause severe consequences in immunodeficient individuals or increase the risk of acquiring other pathogens (28). Thus, the effects of viral infection on the fitness of YFPs, including viral reactivation and possible complications, need further investigation.

Previously discovered PVs in the Cetaceans have been identified within the genera *Omikron-*, *Upsilon-*, and *Dyopipapillomavirus*. Without exception, newly identified PVs in YFPs (NaPVs) can be classified in these three genera based on the phylogenetic trees derived from concatenated E1, E2, L2, and L1 nucleotide sequences. According to species demarcation criteria in individual genera, two novel species (referred to as *Omikronpapillomavirus 2* and *3*) and four novel isolates of PVs from YFPs were proposed. Based on the phylogenetic trees derived from concatenated E1, E2, L2, and L1 nucleotide sequences, *Omikron-*, *Upsilon-*, and *Dyopipapillomavirus* are grouped with *Alpha*- and *Omegapapillomavirus* by forming a large crown group (Fig. 4B). They also cluster with *Alphapapillomavirus* according to the phylogenetic trees based on the early genes E1 and E2 (Fig. 5B). In general, the topology of the phylogenetic trees derived from the early genes predominantly reflects the niche adaptation of PVs (29). Considering both their topology in the phylogenetic trees and the isolation source, we suggest mucosal tropism of NaPVs and other cetacean PVs. Because of the diverse genome and the multiple infections in close relation with other cetacean PVs in the phylogenetic trees, NaPVs were inferred as mucosal low-risk viruses. We are less certain whether NaPVs can be considered parts of the normal microbiome, since many human skin PVs display a commensal nature due to their unique genomic diversity (30).

PVs are considered ancient viruses that have, for the most part, coevolved with their hosts (16). Increasing evidence indicated that they did not follow strict coevolution with their hosts, as exemplified by PVs belonging to divergent genera identified in the same hosts. Similarly, NaPVs, the newly identified PVs, in YFPs were also proposed to be classified into three different genera. Evolution profile analysis revealed the ancient pathway of evolutionary events, including recombination, duplication, and transfer, in cetacean PVs (31, 32). As well, these evolutionary events are also detected using multiple algorithms, although cospeciation mainly drives papillomavirus evolution (Fig. 7). A previous report suggested that TtPV1 and TtPV3 share an ancestor that was generated via recombination of an ancestor of PsPV1, which infected *T. truncates* and Phocoena spinipinnis (17). Our analyses indicate that NaPV2 was generated via recombination of an ancestor of TtPV1 and PsPV1. Surprisingly, transfer infection events were also inferred between *T. truncates* and YFPs and between *T. truncates* and *Lagenorhynchus acutus*. Both the recombination and transfer events among these PVs imply that their hosts have overlapping niches. According to Fahrenholz’s rule, the evolutionary history of a pathogen could mirror that of its host, both in divergence times and phylogenic history, in scenarios of coevolution (33).

Two molecular clock models, including the relaxed-clock log-normal model and TDRexp model, were used to evaluate the divergence times of cetacean PVs, including NaPVs (Fig. 8 and Fig. S1). The divergence times between two given PVs based on the TDRexp clock model were later than those obtained with the relaxed-clock log-normal model. In accordance with previous reports, the TDRexp model might not suitable for cetacean PVs considering the inconsistent phylogenetic tree topologies of early and late genes and the presence of multiple potential recombination events in cetacean PVs (31, 32, 34). Based on the relaxed-clock log-normal model, the majority of cetacean PVs split around the second period of ocean restructuring (13 to 4 Mya), reflecting the diversifying radiation of cetacean species within the *Delphinidae* and *Phocoenidae* (35). Fossils revealed that Cetacea originated 56 to 53 Mya from terrestrial artiodactyl ancestors, which was supported by the divergence time of SsPV and their common ancestors of *Dyopipapillomavirus 1* (52.7 Mya). Currently, PVs have been identified from only 5 species within *Delphinidae* and *Phocoenidae*. Discovery of more viruses, especially the conserved polyomaviruses, herpesviruses, or PVs from cetaceans, could reflect the phylogeny of their hosts and remedy the deficiency of fossil evidence for cetaceans.

## MATERIALS AND METHODS

### Sample collection.

YFPs were collected in April 2021 in the TEO reserve using the method of sound chase and net capture, as described previously (36). Gender, body weight, and body length were recorded individually (Table S2). A total of 58 fecal swabs were obtained, immersed in maintenance medium individually, and kept in liquid nitrogen before transfer to a −80°C freezer for storage.

### RAN extraction, library preparation, and sequencing.

Five fecal swabs were pooled as one sample for library preparing and sequencing (37, 38). Viral RNA from each pool was isolated using TRIzol LS reagent (Thermo Fisher Scientific). Metatranscriptome library preparation of each pool was carried out using the TruSeq stranded total RNA sample preparation kit from Illumina (San Diego, CA) after removal of host rRNA using Illumina Ribo-Zero rRNA removal kits (San Diego, CA). Paired-end (150-bp) sequencing of each RNA library was performed using the DNBSEQ-T7 platform according to the manufacturer’s instructions. Library preparation and sequencing were performed by Magigene (Guangzhou, China).

### Virus discovery and confirmation.

For each library, sequencing reads were first quality controlled using bbduk.sh (https://sourceforge.net/projects/bbmap/; parameters: maq = 10; qtrim = r; trimq = 10; ftl = 1; minlen = 90). The remaining reads were assembled *de novo* using MEGAHIT (39) (version 1.2.8) deploying default parameters. The assembled contigs were compared against the NCBI nonredundant protein database (nr) using Diamond BLASTx (version 0.9.25.26) (40). The E-value cutoff was set at 1E−3 to maintain high sensitivity at a low false-positive rate. Taxonomic lineage information was obtained for the top BLAST hit of each contig, and those classified in the kingdom ‘‘Viruses’’ were identified as probable virus hits. Contigs with unassembled overlaps were merged using the SeqMan program implemented in the Lasergene software package, version 7.1 (DNAstar) (41). The final virus genomes were verified by mapping reads to the corresponding contigs and inspecting the mapping results using Geneious (42). The presence of viruses was further verified using PCR or RT-PCR on the original RNA samples with primer sets designed based on the viral genome sequences. The PCR products were then sequenced and compared with the original sequences. For incomplete viral genomes, the internal gaps were filled by PCR or RT-PCR and Sanger sequencing.

### Estimation of virus prevalence and abundance.

Each virus sequence was first assigned to specific virus species based on the level of sequence similarity to each other and to reference sequences. To estimate the relative abundance of each virus species in each library, quality-trimmed reads were first mapped to the SILVA database (www.arb-silva.de; version 132.1) using Bowtie2 (version 2.3.5.1) (43) to remove reads associated with rRNA. Unmapped reads were subsequently mapped to confirmed viral genomes using the ‘‘end-to-end’’ setting, and the abundance of each virus species was estimated as the number of mapped reads per million total reads (RPM) in each library. To limit false positives, we considered an RPM value greater than or equal to 10 as providing evidence for a positive virus hit.

### Phylogenetic analysis.

Phylogenetic analyses were performed based on the combinations of E1-E2-L2-L1 related amino acid sequences, separate L1 sequences, and E1 nucleotide sequences. Multiple-sequence comparisons were conducted using MUSCLE v 3.8.1551 (44). Reference sequences include 86 papillomaviruses (Table S3). IQ-TREE v 1.6.12 was used to select the most appropriate model based on Bayesian information criteria (E1-E2-L2-L1, LG+F+R9; L1 and E1, GTR+F+R6), and then the phylogenetic trees were estimated by the maximum-likelihood method with 1,000 bootstrap replications (45). The rooting method for the E1-E2-L2-L1, L1, and E1 phylogenetic trees is the midpoint method.

### Recombination analysis.

To detect potential recombination events among cetacean papillomaviruses (Table S4), MUSCLE v3.8.1551 alignments containing all full-length genomic nucleotide sequences of cetacean papillomaviruses were used as input in RDP4 (46), which contains a set of seven recombination detection programs: bootscan, maxchi, chimaera, 3seq, geneconv, lard, and siscan (47–53). The default detection thresholds were used in all cases. Recombination events that were identified by the seven methods were further confirmed using the sliding window method BootScan implemented in Simplot v3.5.1 (54). BootScan analysis was performed with the Kimura (2-parameter) model, using a window size of 300 nucleotides and a step size of 40 nucleotides.

### Positive-selection analysis.

Positive-selection analysis was performed using four codon-based maximum-likelihood methods, namely, single-likelihood ancestor counting (SLAC), fixed-effects likelihood (FEL), evolving mixed effects model (MEME), and fast, unconstrained Bayesian applied inference (FUBAR), in the HYPHY web interface (https://www.datamonkey.org) (55–58). These methods estimate the number of nonsynonymous substitutions (*dN*) and synonymous substitutions (*dS*) for each codon in a multiple alignment. A *dN*/*dS* ratio of >1 indicates positive or diversified selection, a *dN*/*dS* ratio of <1 indicates negative or purifying selection, and if the *dN*/*dS* ratio is 1, neutral evolution can be assumed. For these analyses, multicodon alignments were performed for each gene of cetacean papillomavirus (E6, E1, E2, E4, L2, and L1) using MUSCLE v3.8.1551, and the corresponding predicted protein sequences were aligned. Each alignment included all cetacean papillomaviruses in GenBank, plus those newly identified in this study (Table S5). To minimize the effect of recombination on *dN*/*dS* ratio calculations, recombinant sequences from the recombination analysis described above were removed so that only nonrecombinant sequence partitions were examined, and independent alignments of each papillomavirus gene were analyzed for positive selection signals. The significance level for positive selection results for FEL, SLAC, and MEME analyses was a *P* value of 0.1, and for FUBAR positive selection results, it was a *P* value of 0.9.

### Cophylogenetic analysis.

Divergence between cetacean papillomavirus species and host species was mapped using eMPRess (59), *Dyopi*-, *Omikron*- and *Upsilonpapillomavirus* were generated according to four different cost scenarios (Table S6) to assess solutions for different evolutionary considerations, including codivergence, duplication, loss, and transfer events, with scenario 1 favoring codivergence and assigning costs to duplication and loss events, scenario 2 favoring codivergence and loss events because virus prevalence may have been low at some time in the past, scenario 3 assigning higher costs for lineage duplication and highest costs for host-switching events because there is little evidence for such events, and scenario 4 assigning higher costs and also assigning the highest costs to host conversion events. The complete mitochondrion DNA sequences from the host species and the E6-E1-E2-L2-L1 nucleotide sequences of the papillomavirus concatenation were compared separately using MUSCLE. Phylogenetic trees were constructed separately using MEGA11 (60) by the maximum-likelihood method.

### Estimation of divergence time.

The Bayesian Markov chain Monte Carlo (MCMC) method implemented in BEAST v.1.10.4 (61), and previously published PV evolutionary rates (62) were used to estimate the divergence times of cetacean PVs from their MRCA. Considering the fact that cetacean PVs do not follow a strict virus-host coevolution, divergence times were calculated separately for *Dyopipapillomavirus* (*n* = 3), *Omikronpapillomavirus* (*n* = 7) and *Upsilonpapillomavirus* (*n* = 10) (see the supplemental material). To estimate the three tree-like priors, the demographic Yule model was used, assuming the existence of uncorrelated branches between the rate changes of the relaxed log-normal distribution molecular clock model. In addition, the best-fit model was used separately for concatenated nucleotide sequences using PhyloSuite v1.2.2. The concatenated nucleotide sequence partitions of five ORFs (E6, E1, E2, L2, and L1) were used, and their substitution rates varied over time. There were 2.39 × 10^−8^ (95% confidence interval [CI], 1.70 × 10^−8^ to 3.26 × 10^−8^) substitutions per site per year for the E6 gene, 1.76 × 10^−8^ (95% CI, 1.20 × 10^−8^ to 2.31 × 10^−8^), 2.11 × 10^−8^ (95% CI, 1.52 × 10^−8^ to 2.81 × 10^−8^) for the E2 gene, 2.13 × 10^−8^ (95% CI, 1.46 × 10^−8^ to 2.76 × 10^−8^) for the L2 gene, and 1.84 × 10^−8^ (95% CI, 1.27 × 10^−8^ to 2.35 × 10^−8^) for the L1 gene (18).

To calibrate the divergence times, two time points were introduced within the *Dyopi-*, *Omikron-*, and *Upsilonpapillomavirus* trees, and a common divergence history between the PVs of cetaceans and their hosts was assumed. For the *Dyopipapillomavirus* phylogenetic tree, the calibration time points between *Ursus maritimus papillomavirus 1* (UmPV1) and Sus scrofa *papillomavirus 1* (SsPV1) were at 75 Mya (95% CI, 73.5 to 81.7 Mya) (63), corresponding to the divergence between polar bear and pig ancestors, and those between TtPV8 and PphPV4 were at 17.6 Mya (95% CI, 16.0 to 19.2 Mya) (64), corresponding to the divergence between porpoise and spinner dolphin ancestors. For the *Upsilonpapillomavirus* and *Omikronpapillomavirus* phylogenetic trees, the calibration time point between *Rusa timorensis papillomavirus 1* (RtiPV1) and *Phocoena phocoena papillomavirus 1* (PphPV1) was at 57.5 Mya (95% CI, 55.4 to 59.6 Mya) (65), corresponding to the divergence between the terrestrial and aquatic even-toed ancestors, and another calibration time point was set between the porpoise PV and its close relative, the dolphin PV (64).

MCMC analyses for *Dyopi*-, *Omikron*-, and *Upsilonpapillomavirus* were run for 20,000,000 steps (subsampling every 1,000 generations), 40,000,000 steps (subsampling every 2,000 generations), and 80,000,000 steps (subsampling every 4,000 generations), respectively, setting up the first 10% steps of discard burns to refine the tree and log files for further analysis. The effective sample size (ESS) was evaluated for all parameters using Tracer v.1.7.2 (66), and an ESS value of >400 indicates that all Bayesian chains are well sampled and have converged. FigTree v.1.4.4 was used to visualize the phylogenetic tree (http://tree.bio.ed.ac.uk/software/figtree/).

For comparison, an exponential time-dependent rate (TDRexp) clock model was used to estimate divergence times of the PVs in cetaceans (67, 68). The exponential epoch structure was used and the boundaries of the epochs were set up as 0 < 10^−5^ < 10^−4^ < … < 10^2^ < ∞. The additional calibration time point between RtiPV1 and *Capra hircus papillomavirus 1* (ChPV1) was introduced at 22.8 Mya (95% CI, 18.5 to 27.8 Mya). MCMC analyses for *Dyopi-*, *Omikron-*, and *Upsilonpapillomavirus* were run for 60,000,000 steps (subsampling every 3,000 generations), 60,000,000 steps (subsampling every 3,000 generations), and 90,000,000 steps (subsampling every 3,000 generations), respectively.

### Statistical analysis.

Data were analyzed using two-tailed Student’s tests (for two-group comparisons) or analysis of variance (ANOVA; for multiple-group comparisons). For all tests, statistical significance was assumed when *P* was <0.05.

### Data availability.

The novel viral genomes described in detail here were deposited in GenBank under accession numbers OQ274126 to OQ274131. The raw sequence reads from the viral metagenomic libraries were deposited in the Short Read Archive of GenBank under the accession number PRJNA924090.
