# Supplementary material for: Viral Diversity and Epidemiology in Critically Endangered Yangtze Finless Porpoises (Neophocaena asiaeorientalis asiaeorientalis)
Source: Microbiol Spectr. 2023 Jun 5;11(4):e00810-23. doi: 10.1128/spectrum.00810-23 (PMC10434060; doi:10.1128/spectrum.00810-23)
Supplement: Supplemental file 1 — Fig. S1. Download spectrum.00810-23-s0001.pdf, PDF file, 0.10 MB [file spectrum.00810-23-s0001.pdf]

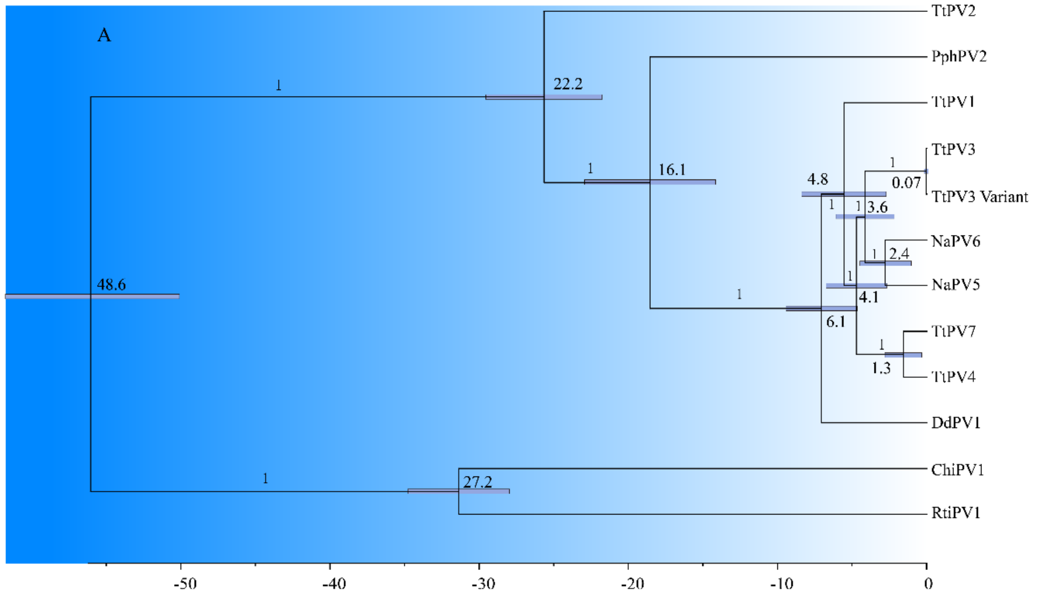

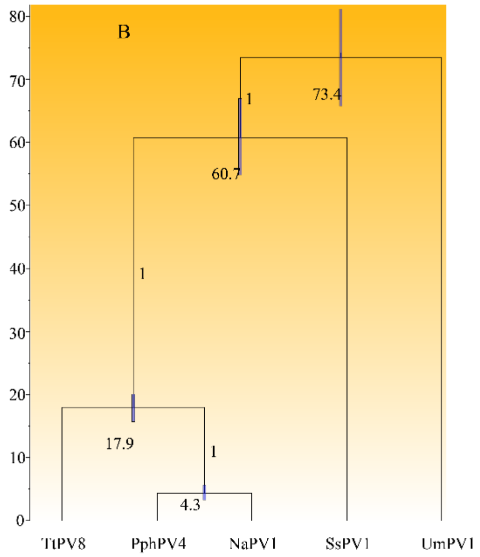

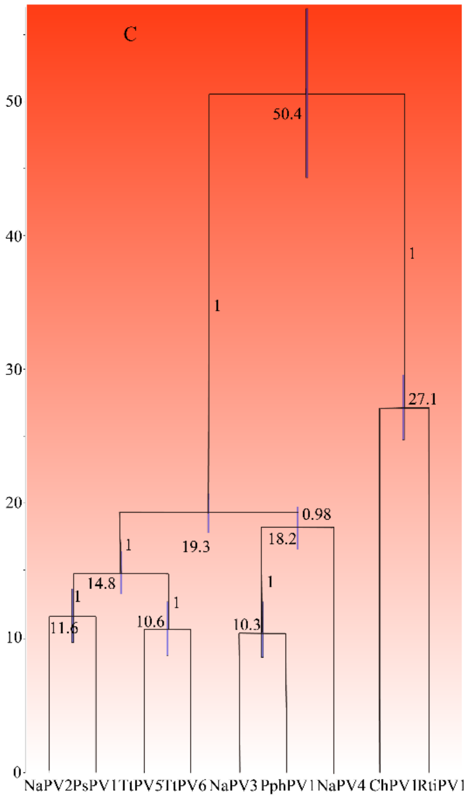

Figure S1. A Bayesian MCMC method was used to estimate divergence times. Times were calculated separately for each genus, *Upslion*- (**A**), *Dyopi*- (**B**) and *Omikron*-PVs (**C**). Numbers above the nodes are the mean estimated divergence time in million years

(Mya). The bars represent the 95% highest posterior density (HPD) interval for the divergence times. Panels B and C show time on the Y-axis and phylogeny on the X axis.
